# Supplementary material for: Evaluation of Pyrophosphate-Driven Proton Pumps in Saccharomyces cerevisiae under Stress Conditions
Source: Microorganisms. 2024 Mar 20;12(3):625. doi: 10.3390/microorganisms12030625 (PMC10976212; doi:10.3390/microorganisms12030625)
Supplement: Supplementary file 1 [file microorganisms-12-00625-s001.zip › microorganisms-2888718-supplementary.pdf]

Supplementary Data

Supplementary Table S1: List of all the primers used for cloning and sequencing in this study.

Primer Name	Primer Sequence
**Cloning Primers**	
88 – Tc_VP_R	5′ - ctcaccccccgccggtg
88r – APV1_FW	5′ - atggttgctccagctttgttgcca
89 – Suc2SP_F	5′ - atgcttttgcaagctttccttttcc
90 – KS_Tef1p_rev	5′ - tttgtaattaaaacttagattagattgctatgctttc
NM_GPDp_Sac1_FW_F	5′ - agctggagctcagtttatcattatca
NM_CYC7tSdaI_RV_R	5′ - aacctgcaggtaccggccgcaaattaaagc
84r – phlu_BamH_F	5′ - acaggatccatgagtaaaggagaagaacttttcac
85 – phlu_EcoRI_R	5′ - gcagaattcttatttgtatagttcatccatgcc
**Sequencing Primers**	
RFP_18_seqADH1t	5′ - tcgcttatttagaagtgtcaac
90r – KS_DeltaTc	5′ - agaggttcatcgtggcg
89r – Seq_R_Avp1	5′ - tctgattctgtgagacataccagc
57 – CYC1_Seq_R	5′ - gcgtgaatgtaagcgtgac
NM_CYC7t_REV_R	5′ - agggcgtgaatgtaagcgtg
NM_GPDp_FW_F	5′ - accttctgctctctctgatttgg
87r – YFPseq_F	5′ - cactaccagcagaacac
LW_262_TDH3p_rev	5′ - atccgtcgaaactaagttctgg
69r – T7_Seq_F	5′ - taatacgactcactataggg
RPF_21_X-4_ver_f	5′ - cgtgcccaaagctaagagtc
LW_99_5'GRE3_r_LWA20	5′ - ctggatgccagcttaaaaag
RFP_24_XI-3_ver_r	5′ - cggttgtgatattgttcctgc
RFP_23_XI-3_ver_f	5′ - ggccgttatttgtgcttgat

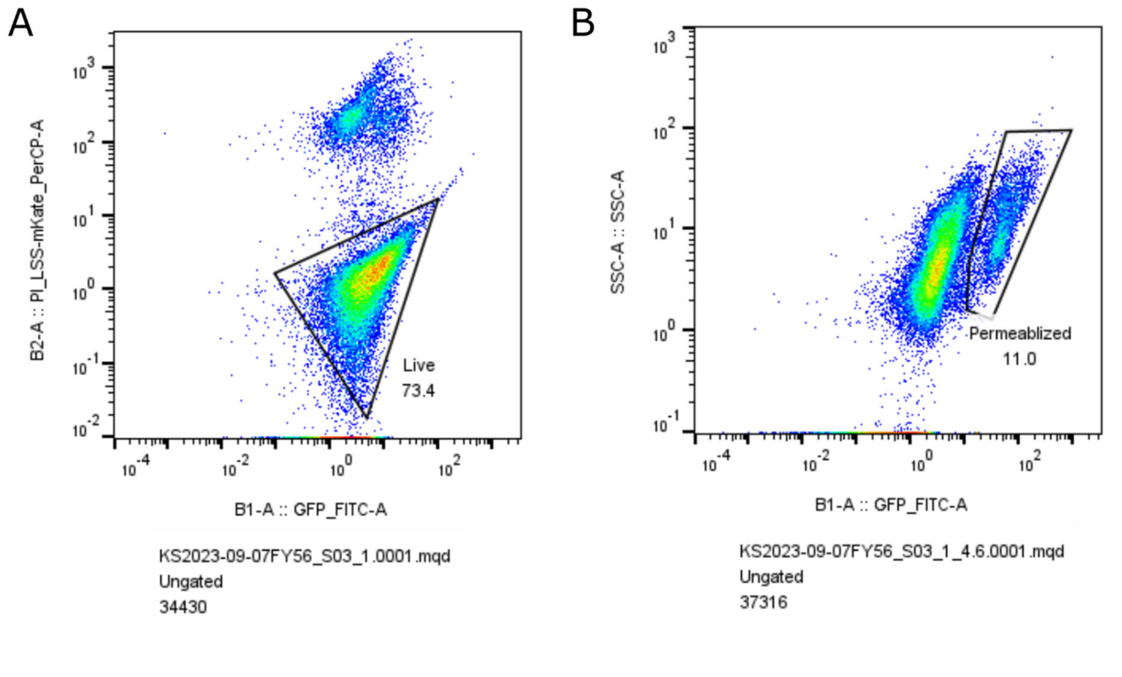

Supplementary Figure S1: Gating strategy for cells stained with pHrodo® green. (A) is the gating strategy to obtain the geometric mean of non-permeable stained cells. (B) is the strategy used to obtain the geometric mean of permeabilised cells for making the standard curve.

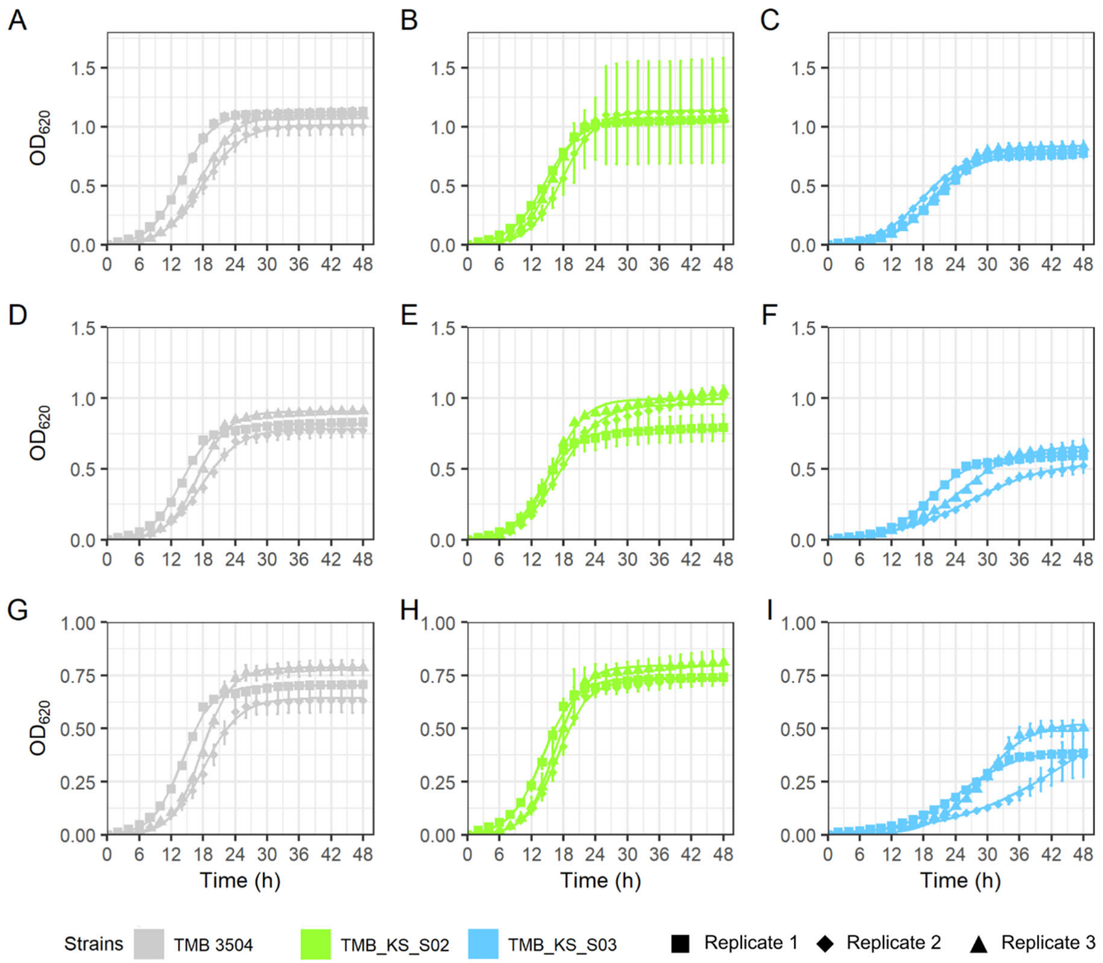

Supplementary Figure S2: Growth curves of the three strains without biosensors in mineral media at pH 5 in microtiter plates. (A), (D) and (G) are the growth profiles of TMB 3504 in 0, 3 and 6 g·L^{-1} of acetic acid, respectively. (B), (E) and (H) are the growth profiles of TMB_KS_S02 in 0, 3 and 6 g·L^{-1} of acetic acid, respectively. (C), (F) and (I) are the growth profiles of TMB_KS_S03 in 0, 3 and 6 g·L^{-1} of acetic acid, respectively. The square, diamond and the triangle shapes are biological replicates with three technical replicates (3 individual wells inoculate from separate colonies obtained from a single clone) represented as standard deviations for each biological replicate. The solid lines are the logistic models fitted through the technical replicates for each biological replicate.

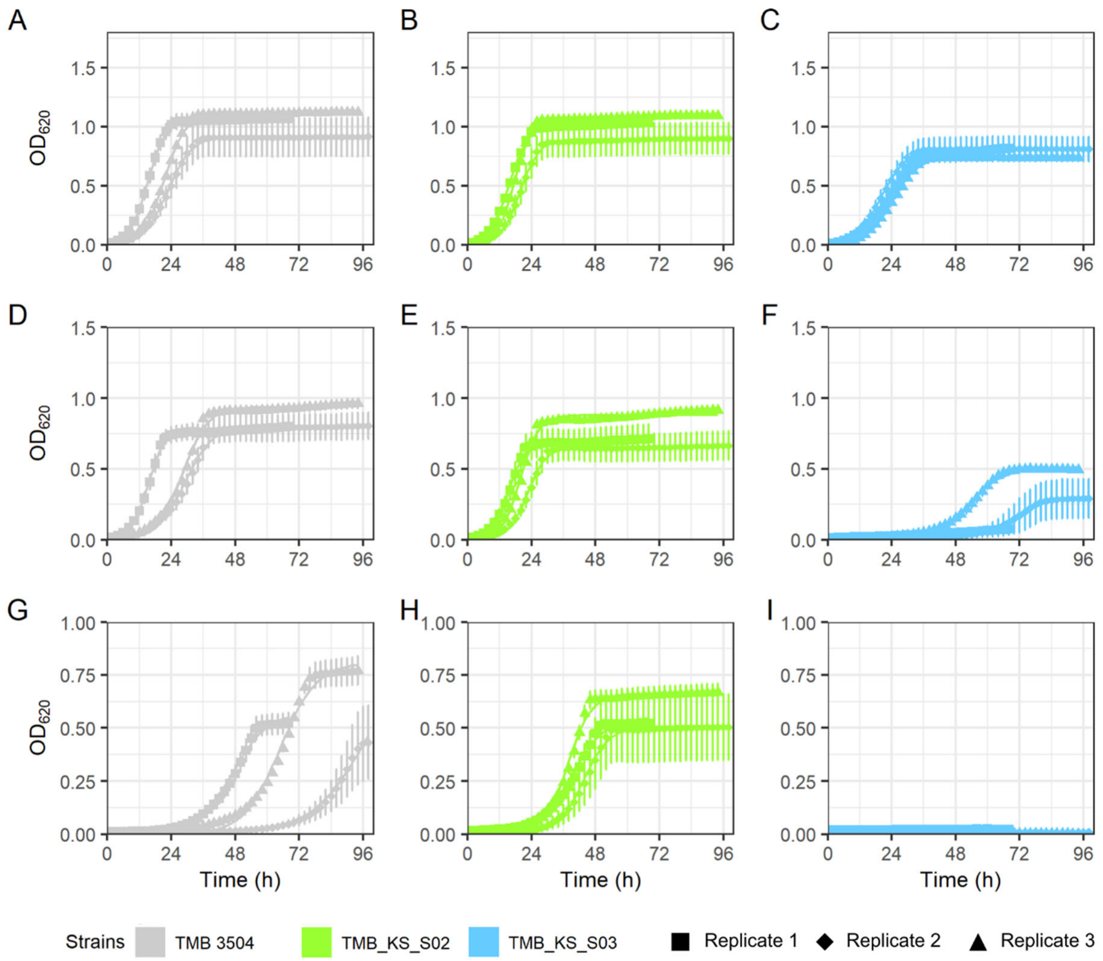

Supplementary Figure S3: Growth curves of the three strains without biosensors in mineral media at pH 3.7 in microtiter plates. (A), (D) and (G) are the growth profiles of TMB 3504 in 0, 3 and 6 $g \cdot L^{-1}$ of acetic acid, respectively. (B), (E) and (H) are the growth profiles of TMB_KS_S02 in 0, 3 and 6 $g \cdot L^{-1}$ of acetic acid, respectively. (C), (F) and (I) are the growth profiles of TMB_KS_S03 in 0, 3 and 6 $g \cdot L^{-1}$ of acetic acid, respectively. The square, diamond and the triangle shapes are biological replicates with three technical replicates (3 individual wells inoculate from separate colonies obtained from a single clone) represented as standard deviations for each biological replicate. The solid lines are the logistic models fitted through the technical replicates for each biological replicate.

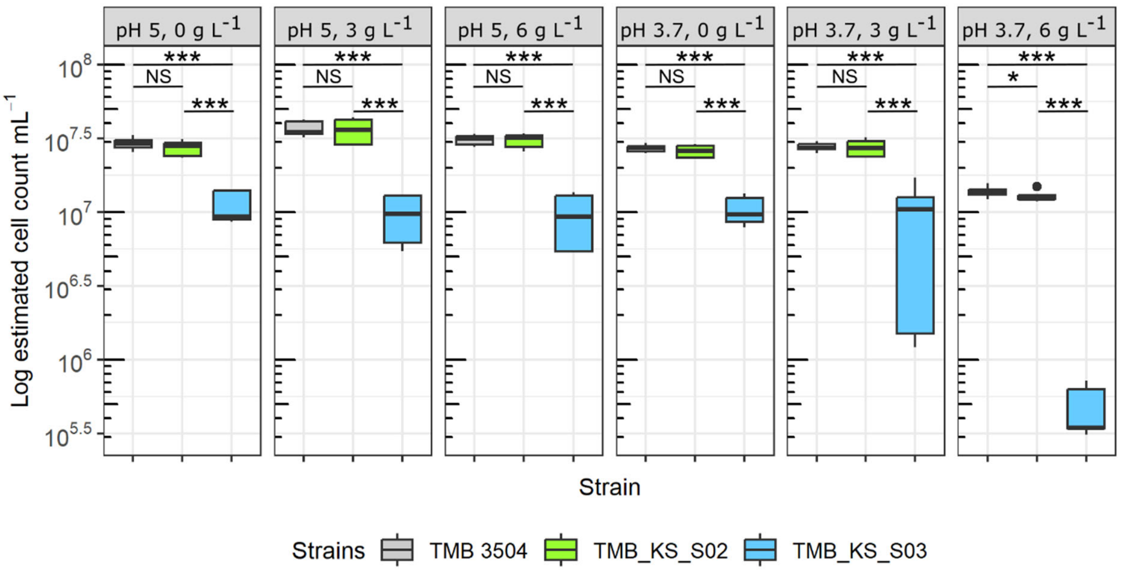

Supplementary Figure S4: Estimated log_{10} of the total cell count per mL calculated using flow cytometry at the end point for the parent strain (TMB 3504), Vacuolar H^+-PPase strain (TMB_KS_S02) and the cell membrane H^+-PPase strain (TMB_KS_S03) in all the different concentrations of acetic acid in minimal media at different pH 5 and 3.7. [The box represents the quartiles from a combination of technical and biological replicates (9 individual wells), outliers are represented as dots. (NS) represents a p-value greater than 0.1, (*) represents a p-value between 0.01 and 0.05, (***) represents a p-value between 0 and 0.001.] [ANOVA p-values for the various conditions are as follows (pH 5 (0 $g \cdot L^{-1}$, $P = 1.56 \times 10^{-13}$; 3 $g \cdot L^{-1}$, $P = 3.72 \times 10^{-12}$; 6 $g \cdot L^{-1}$, $P = 6.74 \times 10^{-14}$), pH 3.7 (0 $g \cdot L^{-1}$, $P = 2.29 \times 10^{-15}$; 3 $g \cdot L^{-1}$, $P = 7.55 \times 10^{-10}$; 6 $g \cdot L^{-1}$, $P = 2 \times 10^{-16}$))]

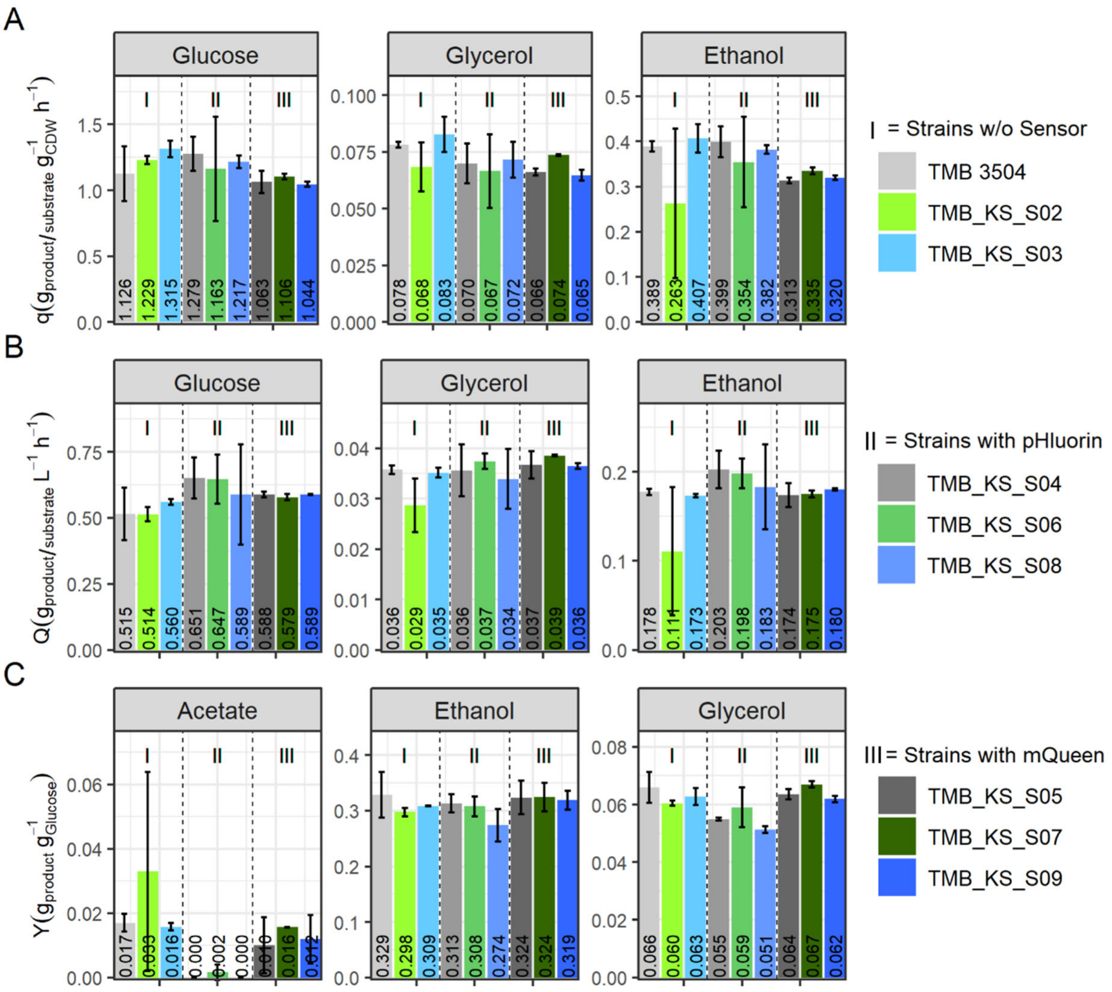

Supplementary Figure S5: Specific productivity (q), volumetric productivity (Q) and yield (Y) of the various strains grown in 20 g·L^{-1} glucose in bioreactors. [Standard deviations between replicates are represented as error bars]. (A) shows the q_{xylose}, $q_{xylitol}$ and $q_{ethanol}$ calculated during logarithmic growth. (B) shows Q_{xylose}, $Q_{xylitol}$ and $Q_{ethanol}$ calculated during logarithmic growth. (C) shows the Y_{xylose}, $Y_{xylitol}$ and $Y_{ethanol}$ calculated for the entire duration of the fermentation. TMB 3504 is the parent strain, and TMB_KS_S02 and TMB_KS_S03 are its derivatives with the proton pump targeted to the vacuolar and cytosolic membrane, respectively. TMB_KS_S04, TMB_KS_S06 and TMB_KS_S08 are the derivatives of TMB_3504, TMB_KS_S02 and TMB_KS_S03, respectively, with the pHluorin biosensor. TMB_KS_S05, TMB_KS_S07 and TMB_KS_S09 are the derivatives of TMB_3504, TMB_KS_S02 and TMB_KS_S03, respectively, with the QUEEN-2m biosensor.

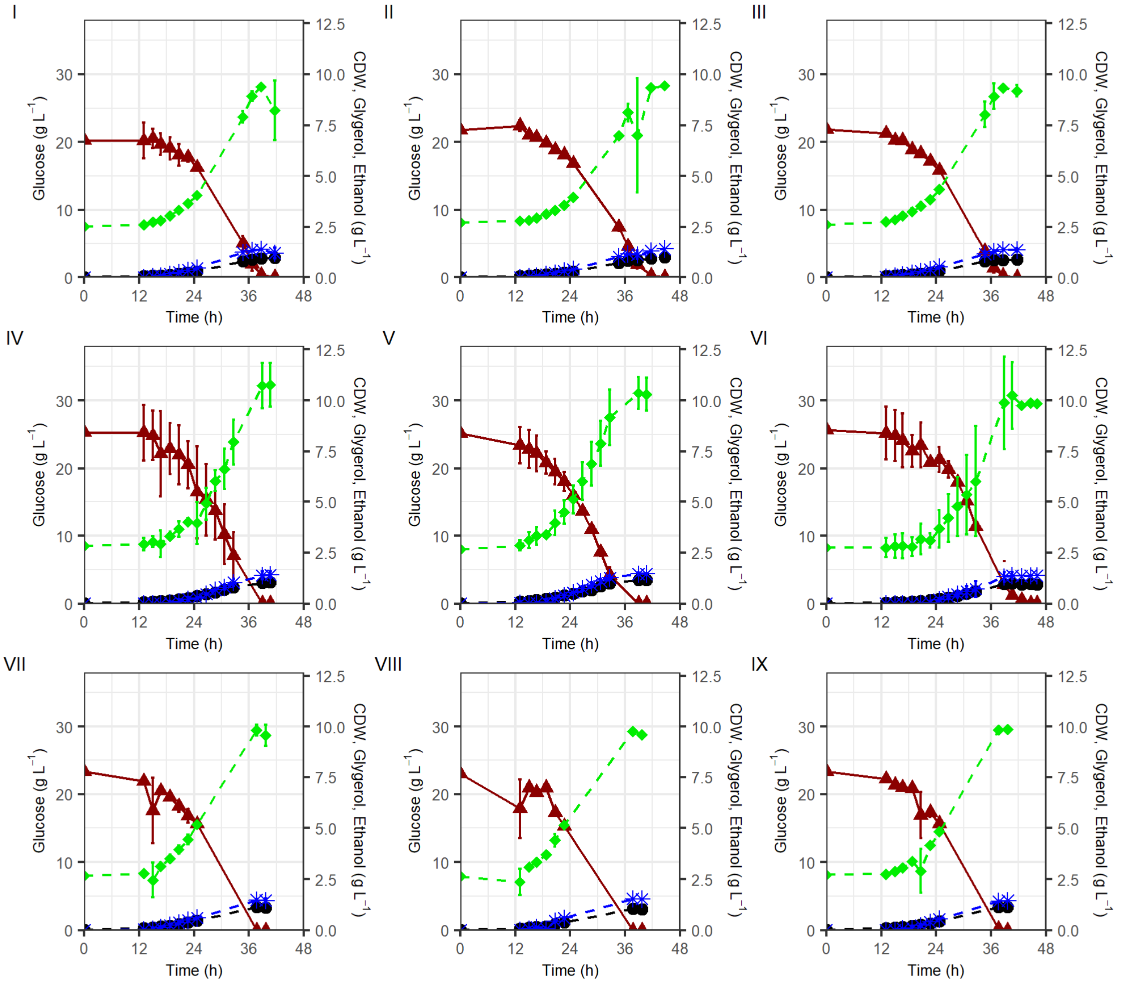

Supplementary Figure S6: Metabolic profiles of the strains grown anaerobically on 20 g·L^{-1} glucose. [I] is the parent strain (TMB 3504), [II] is the vacuolar membrane H^{+}-PPase strain (TMB_KS_S02), [III] is the cell membrane H^{+}-PPase strain (TMB_KS_S03), [IV] is the parent strain with pHluorin (TMB_KS_S04), [V] is the vacuolar membrane H^{+}-PPase strain with pHluorin (TMB_KS_S06), [VI] is the cell membrane H^{+}-PPase strain with pHluorin (TMB_KS_S08), [VII] is the parent strain with mQueen (TMB_KS_S05), [VIII] is the vacuolar membrane H^{+}-PPase strain with mQueen (TMB_KS_S07), [IX] is the cell membrane H^{+}-PPase strain with mQueen (TMB_KS_S09). Triangle (dark red continuous line) glucose; Diamond (dark green, dashed line) ethanol; asterix (blue, dashed line) glycerol, circle (black, dashed line) cell dry weight. The error bars are the standard deviations obtained from biological duplicates.

Supplementary Table S2: Carbon and redox balances for all anaerobic fermentations on glucose and xylose conducted in 1-L working volumes in 3 L Applikon bioreactors.

	Glucose		*Xylose*	
	Carbon balance	Redox Balance	Carbon balance	Redox Balance
TMB 3504	1.007 ± 0.149	1.009 ± 0.140	1.035 ± 0.031	1.031 ± 0.020
TMB_KS_S02	0.951 ± 0.049	0.956 ± 0.044	0.993 ± 0.011	1.007 ± 0.039
TMB_KS_S03	0.938 ± 0.088	0.945 ± 0.083	0.980 ± 0.021	0.985 ± 0.029
TMB_KS_S04	0.898 ± 0.079	0.907 ± 0.070	1.034 ± 0.021	1.035 ± 0.017
TMB_KS_S05	0.892 ± 0.106	0.901 ± 0.094	0.995 ± 0.006	0.992 ± 0.010
TMB_KS_S06	0.903 ± 0.091	0.911 ± 0.094	0.987 ± 0.011	0.991 ± 0.022
TMB_KS_S07	0.950 ± 0.100	0.954 ± 0.090	1.007 ± 0.018	1.001 ± 0.025
TMB_KS_S08	0.880 ± 0.067	0.890 ± 0.060	1.053 ± 0.037	1.048 ± 0.033
TMB_KS_S09	0.938 ± 0.110	0.941 ± 0.100	N.D.	N.D.

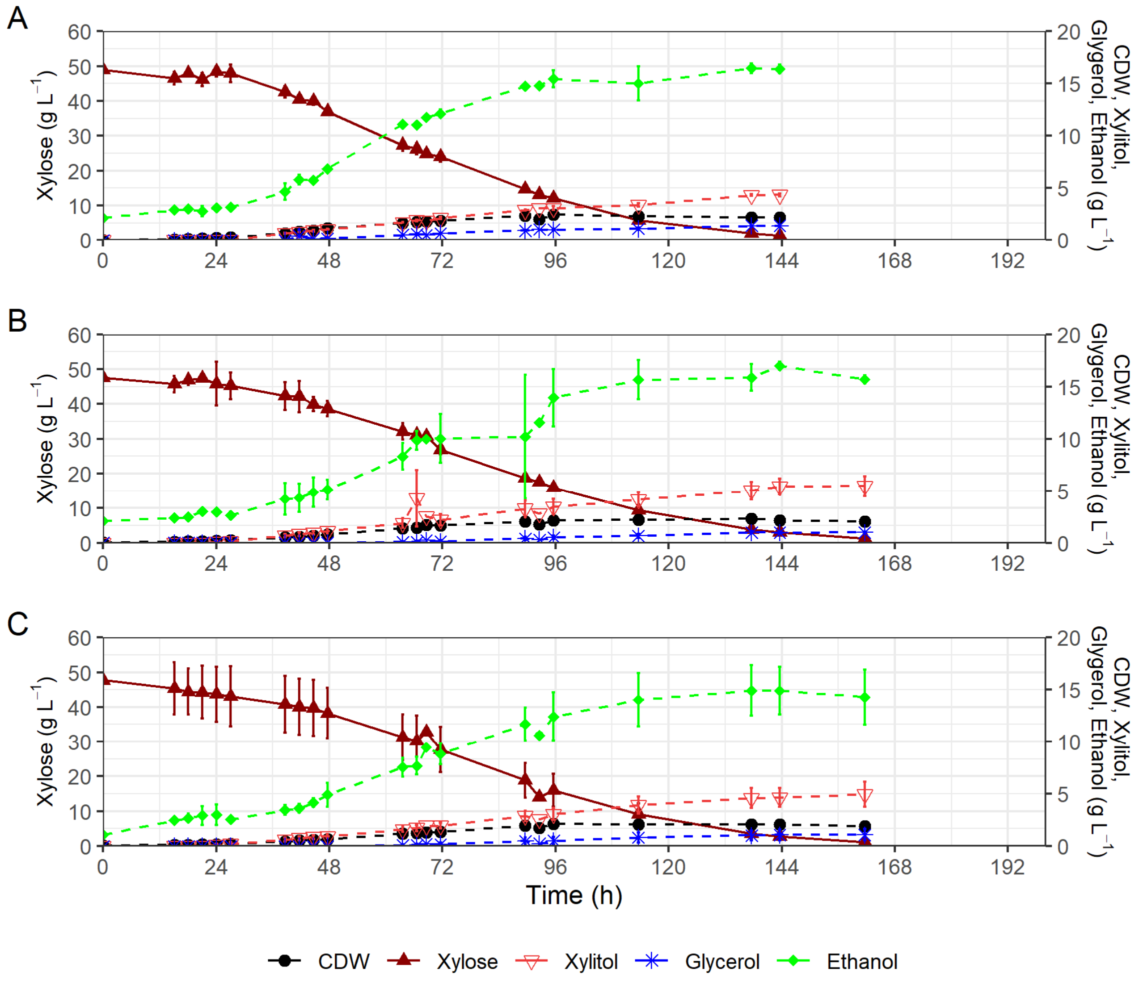

Supplementary Figure S7: Metabolic profiles of the strains grown anaerobically on 50 g·L^{-1} xylose. (A) is the parent strain (TMB 3504), (B) is the vacuolar membrane H^+-PPase strain (TMB_KS_S02), (C) is the cell membrane H^+-PPase strain (TMB_KS_S03). Triangle (dark red continuous line) xylose; inverted triangle (brown, dashed line, no fill) xylitol; diamond (green, dashed line) ethanol; asterix (blue, dashed line) glycerol, circle (black, dashed line) cell dry weight. The error bars are the standard deviations obtained from biological duplicates.

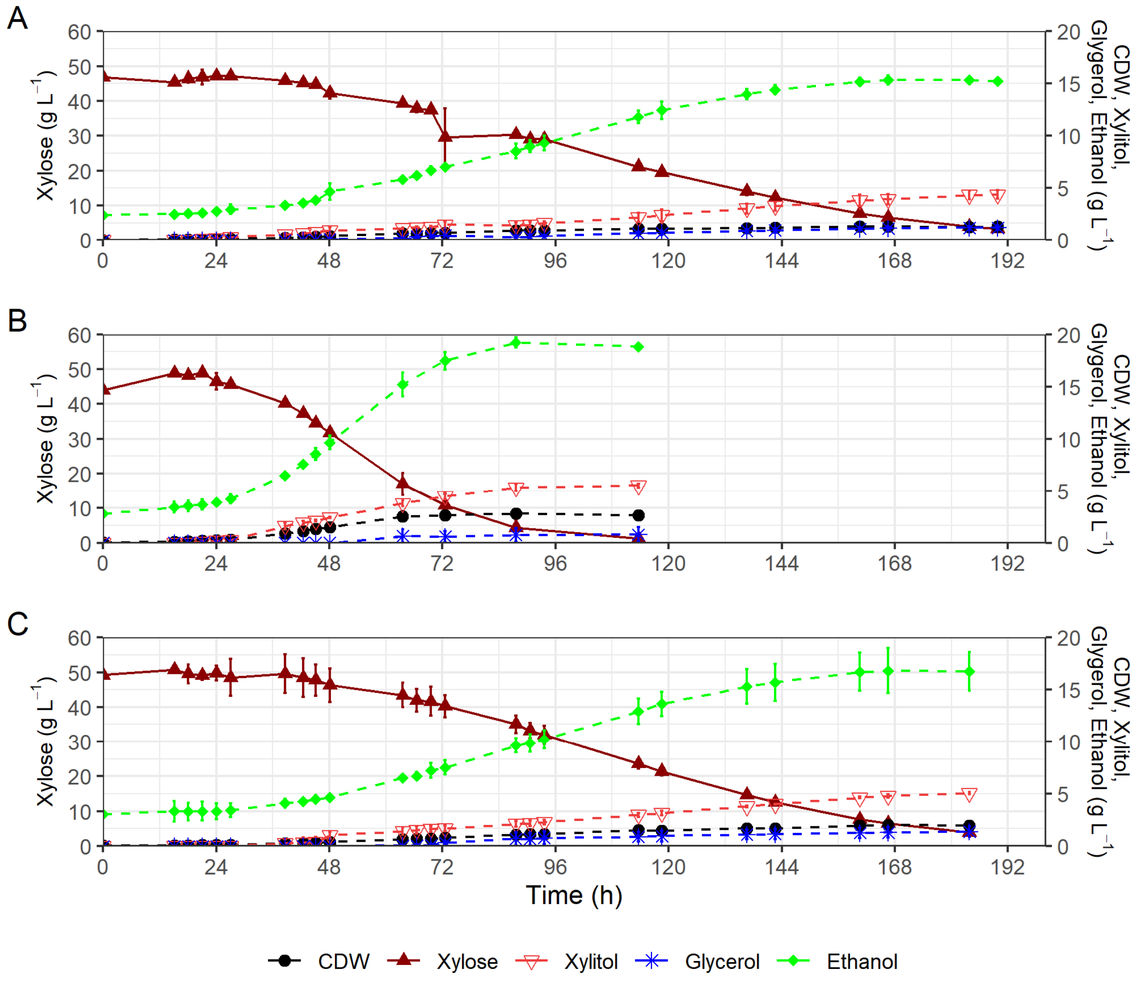

Supplementary Figure S8: Metabolic profiles of the strains grown anaerobically on 50 g·L^{-1} xylose. (A) is the parent strain with pHluorin (TMB_KS_S04), (B) is the vacuolar membrane H^+-PPase strain with pHluorin (TMB_KS_S06), (C) is the cell membrane H^+-PPase strain with pHluorin (TMB_KS_S08). Triangle (dark red continuous line) xylose; inverted triangle (brown, dashed line, no fill) xylitol; diamond (green, dashed line) ethanol; asterix (blue, dashed line) glycerol, circle (black, dashed line) cell dry weight. The error bars are the standard deviations obtained from biological duplicates.

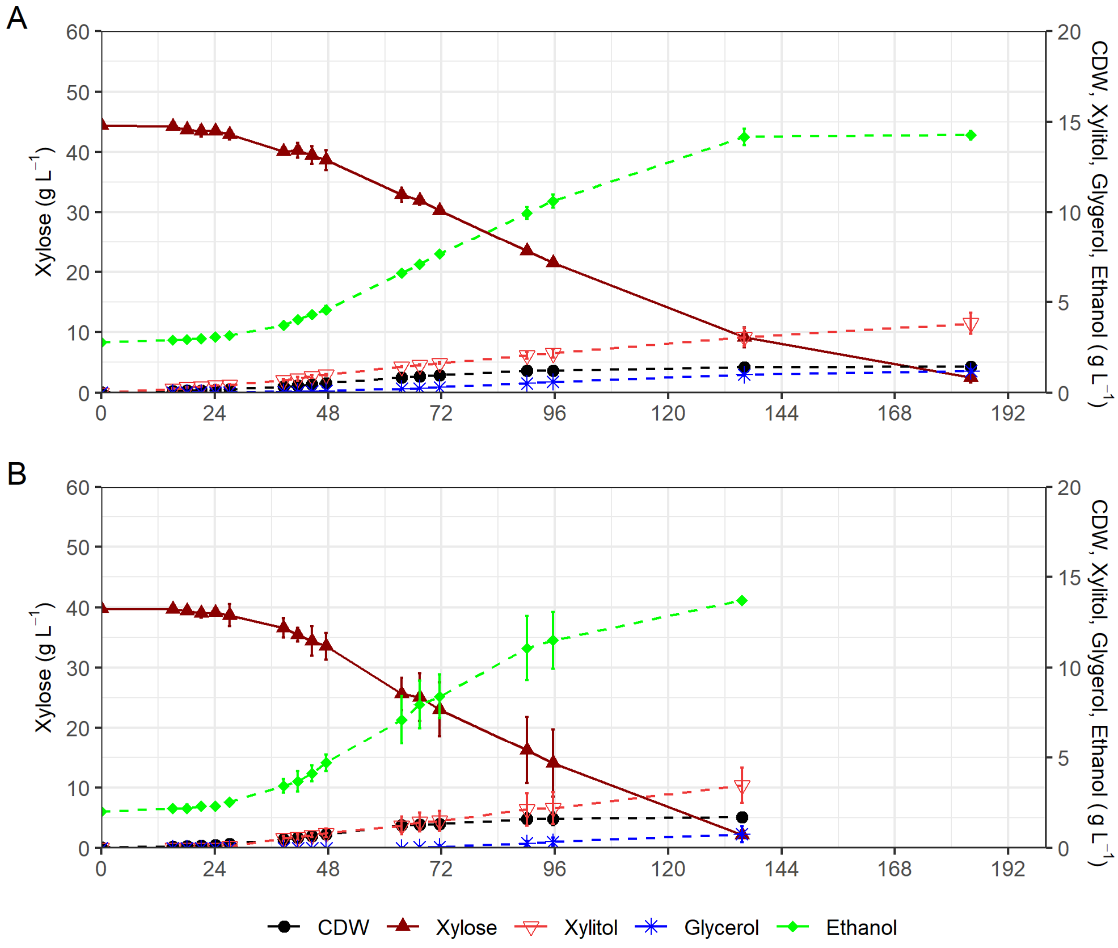

Supplementary Figure S9: Metabolic profiles of the strains grown anaerobically on 50 g·L^{-1} xylose. (A) is the parent strain with mQueen (TMB_KS_S05), (B) is the vacuolar membrane H^{+}-PPase strain with mQueen (TMB_KS_S07). Triangle (dark red continuous line) xylose; inverted triangle (brown, dashed line, no fill) xylitol; diamond (green, dashed line) ethanol; asterix (blue, dashed line) glycerol, circle (black, dashed line) cell dry weight. The error bars are the standard deviations obtained from biological duplicates.

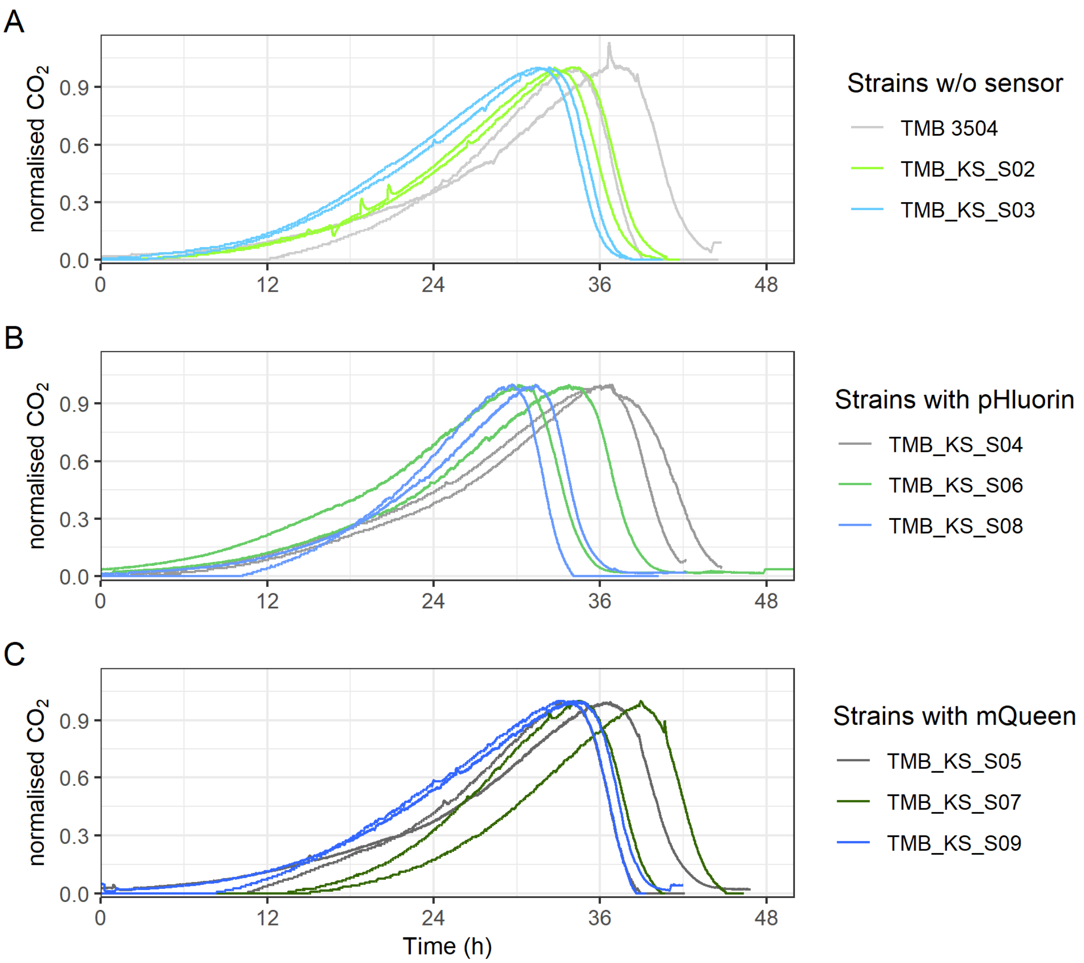

Supplementary Figure S10: Time course of the carbon dioxide production profiles for the biological duplicates for all fermentations on 20 $g{\cdot}L^{-1}$ glucose. Data is normalised to the highest value within their respective fermentations. [A] The parent strain, The vacuolar membrane H^+-PPase strain and the cell membrane H^+-PPase strain, [B] the derivatives of [A] with pHluorin biosensor, [C] derivatives of [A] with mQueen biosensor.

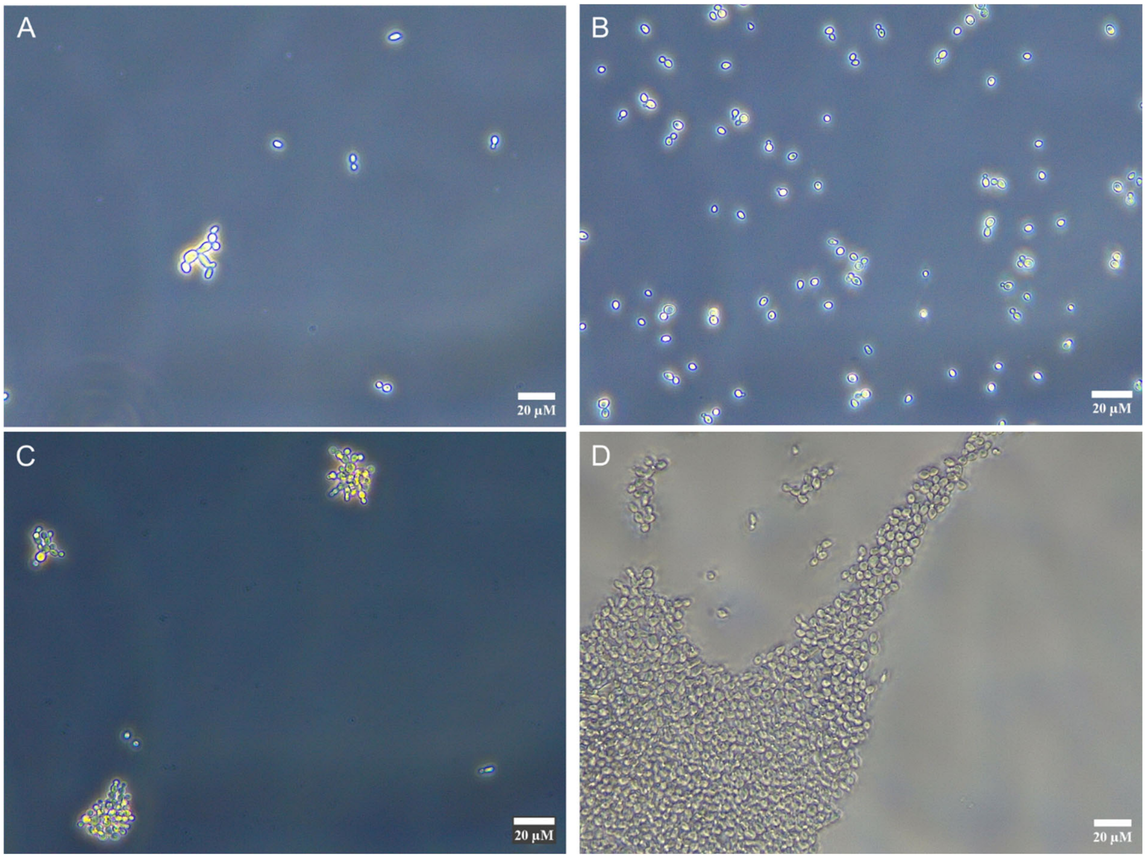

Supplementary Figure S11: Phase contrast micrographs at 40X magnification of the strains with pHluorin biosensor. Images taken during logarithmic growth. (A) Parent strain with pHluorin (TMB_KS_S04), (B) Vacuolar H^+-PPase strain with pHluorin (TMB_KS_S06), (C) Cell membrane H^+-PPase strain with pHluorin (TMB_KS_S08) and (D) Parent strain with mQueen (TMB_KS_S05).
